# Risk for ASD in Preterm Infants: A Three-Year Follow-Up Study

**DOI:** 10.1155/2018/8316212

**Published:** 2018-11-11

**Authors:** Ayelet Harel-Gadassi, Edwa Friedlander, Maya Yaari, Benjamin Bar-Oz, Smadar Eventov-Friedman, David Mankuta, Nurit Yirmiya

**Affiliations:** ^1^School of Education, The Hebrew University of Jerusalem, Mount Scopus, Jerusalem, 91905, Israel; ^2^Department of Psychology, The Hebrew University of Jerusalem, Mount Scopus, Jerusalem, 91905, Israel; ^3^Department of Neonatology, Hadassah University Hospital, Jerusalem, 91120, Israel; ^4^Department of Obstetrics & Gynecology, Hadassah University Hospital, Jerusalem, 91120, Israel

## Abstract

**Background:**

The aim of this study was to examine the long-term risk for autism spectrum disorders (ASD) in individuals who are born preterm and full-term using both observational instruments and parental reports. Neonatal risk factors and developmental characteristics associated with ASD risk were also examined.

**Method:**

Participants included 110 preterm children (born at a gestational age of ≤ 34 weeks) and 39 full-term children assessed at ages 18, 24, and 36 months. The Autism Diagnostic Observation Schedule, the Modified Checklist for Autism in Toddlers, the Autism Diagnostic Interview-Revised, the Social Communication Questionnaire, and the Mullen Scales of Early Learning were administered.

**Results and Conclusions:**

The long-term risk for ASD was higher when parental reports were employed compared to observational instruments. At 18 and 24 months, a higher long-term risk for ASD was found for preterm children compared to full-term children. At 36 months, only one preterm child and one full-term child met the cutoff for ASD based on the ADOS, yet clinical judgment and parental reports supported an ASD diagnosis for the preterm child only. Earlier gestational age and lower general developmental abilities were associated with elevated ASD risk among preterm children.

## 1. Introduction

Autism spectrum disorder (ASD) is a major neurodevelopmental disorder characterized by persistent deficits in social interaction and communication as well as restrictive and repetitive patterns of behavior, interests, or activities [1]. Researchers document that, due to early brain plasticity, intensive early intervention programs can improve cognitive and language abilities as well as adaptive behavior in children with ASD ([2–4]). A growing focus is thus given to identify prodromal and preclinical signs or indicators that are present very early in life among infants who are later diagnosed with ASD [5]. Early signs of ASD have been studied with retrospective parental reports [6, 7] and the analyses of home videos of children who were later diagnosed with ASD [8], with prospective population screening studies of infants who scored positive on early ASD screeners [9, 10] and by longitudinal studies of children in “high-risk” populations for ASD, such as the young siblings of children with ASD [11–13]. In addition to genetic risk factors for ASD, environmental factors also contribute to the risk of ASD. Identified environmental factors include advanced parental age, birth complications, and pregnancy-related factors such as maternal obesity, maternal diabetes, caesarian section, and perinatal exposure to Oxytocin [14, 15]. Premature birth, which is the focus of the current paper, is an additional identified risk factor for ASD [16].

The prevalence of ASD has been estimated as 0.6-2.46% among the general population [17–21]. An increased prevalence of ASD risk (1.8%–41%) has been documented among children who were born preterm (PT) [16, 22–24], emphasizing the need for the early identification of ASD risk among PT cohorts. We previously reported a prevalence of 21% of ASD risk at 8 months, which decreased to 9% at 12 months, using the Autism Observation Scale for Infants [25], and to 8% at 18 months, using the Autism Diagnostic Observation Schedule-Toddler Module (ADOS-T) [26]. Considering the importance of repeated assessments when screening for ASD during the first years of life [27], the current study focuses on follow-up assessments using gold-standard measures at 24 and 36 months.

Whereas the association between premature birth and ASD has been documented, there is heterogeneity in the reported prevalence of ASD among children born PT, and inconsistent findings regarding the prevalence were reported [22]. The inconsistent findings may be due to variations in the inclusion criteria, age of examination, and screening instruments that researchers use. They may also result from the difficulty in differentiating early in life between ASD and other developmental disorders and/or difficulties. For example, PT children are at risk for neurodevelopmental difficulties and disabilities, including cognitive, language, regulation, attention, and motor impairments [16]. These impairments often overlap with early prodromal symptoms of ASD [5], which make the differential diagnosis somewhat more complicated.

Most researchers employ parental screening report questionnaires or diagnostic interview assessment tools, whereas the use of direct observational assessments is somewhat less frequent, although recently more and more common. Among samples of PT toddlers, the rate of positive screening using parental report questionnaires ranged from 10% to 41% [28, 29]. These rates were significantly reduced if children with sensory-motor difficulties and/or cognitive impairments were excluded and when follow-up interviews were conducted [23, 30–32].

Few researchers examined the prevalence of ASD by the ADOS, which is a clinical observational instrument. Developmentally, among samples of young children born PT, the estimated prevalence rate of ASD ranges from 1.8% to 12.9%, using the ADOS [23, 24]. Among samples of adolescents born PT, the estimated prevalence rate of ASD was 7.1%, using the ADOS [33]. These results suggest once more that children born premature have an increased risk of ASD diagnoses, yet the use of observational instruments yield lower rates of ASD diagnoses compared to parental reports. Furthermore, in most of the aforementioned studies, the ADOS was administrated only to PT children who screened positive for ASD using the Modified Checklist for Autism in Toddlers (M-CHAT) and only at one time point. In the current longitudinal study, we innovatively employed the ADOS as well as parental reports at the ages of 18, 24, and 36 months to PT and full-term (FT) children who previously screened positive or negative for ASD, in order to examine high- and low-risk status over time.

### 1.1. Study Rationale

The aim of the current study was to examine long-term risk for ASD among children who were born PT and FT, using a variety of measures, including observational instruments and parental reports for all enrolled participants. The study included PT children born between 24 and 34 weeks of gestational age (GA) and FT children born between 37 and 41 weeks of GA, all of whom were assessed at the ages of 18, 24, and 36 months.

Using parental reports (Autism Diagnostic Interview-Revised (ADI-R) [34], the M-CHAT and the Social Communication Questionnaire (SCQ)) [34], direct clinical assessment with the ADOS, and best clinical judgment (AHG, MY, EF), we set out to evaluate the stability of ASD risk over time examining similarities and differences in ASD risk status as determined by parental reports and direct assessments of trained professionals. Contrary to previous studies (in which follow-up assessments were conducted only for children who screened positive), our study included follow-up assessments for children who screened negative as well. This was done to examine the stability of both high- and low-risk status over time. Finally, we examined neonatal risk factors and developmental characteristics associated with ASD risk. We hypothesized an increased risk for ASD in PT children compared to FT children and that the risk would be higher when using parental reports than when using the ADOS at 18 and 24 months. We further hypothesized that earlier GA and lower general developmental abilities would be associated with ASD characteristics and risk concern among PT children.

## 2. Methods

### 2.1. Participants and Procedure

The participants included 110 PT children (47 girls, 63 boys) and 39 FT children (17 girls, 22 boys). The study was approved by the hospital's Institutional Review Board committee (249–09). Preterm children born at 24–34 weeks of gestation were recruited from the neonatal intensive care unit between 2009 and 2013. Full-term children born at 37–41 weeks of gestation with a birthweight > 2500 g following normal labor and delivery were recruited from the nurseries of the same hospital. The response rates were 52% among the PT group and 20% among the FT group. Refusals were usually due to time constraints or unwillingness to commit to multiple developmental assessments. No significant differences emerged between those who declined participation and those who agreed regarding birth weight and gestational age at birth, thus suggesting that the sample is a representative one. Parents who agreed to participate signed an informed consent form and completed a demographic questionnaire. During the course of the study, three infants who were eventually diagnosed with severe sensory-motor impairments (i.e., cortical blindness and severe cerebral palsy) were excluded from the reported sample. Some children dropped out or missed a single assessment. Complete data was ultimately available for 101 PT and 37 FT children at 18 months, 97 PT and 37 FT children at 24 months, and 94 PT and 33 FT children at 36 months. Demographic information and neonatal medical characteristics are presented in Table 1. There were no significant differences between the PT and the FT groups in the demographic characteristics. As expected, there were significant differences regarding the medical characteristics (all p values <.01).

Data regarding pregnancy, delivery, and postnatal hospitalization were obtained from computer-based hospital records. The assessments, which were conducted in the research laboratory or the child's home at the corrected ages of 18, 24, and 36 months, included ASD and developmental evaluations. In addition, semi-structured developmental interviews were conducted with the parents about children's development, their adaptation to kindergarten, their social abilities and treatments, and parental concerns regarding their child's development. Families were reimbursed for travel costs and provided with a video and report of the assessments.

### 2.2. Measures

#### 2.2.1. Modified Checklist for Autism in Toddlers

The MCHAT [35] is a widely used parental screening questionnaire that consists of 23 yes/no items. It is employed to screen infants between the ages of 16 to 30 months for early signs of ASD. The MCHAT items address sensory responsiveness, early language and communication, social relatedness, and early joint attention. It contains 6 critical and 17 noncritical items. The critical items were identified by a discriminant factor analysis of data derived from children with and without a disorder on the autism spectrum; they include items concerning joint attention (e.g., pro-declarative pointing, bringing to show, following a point), interest in other children, responding to one's own name and imitation. A “failed screening” is defined by a parental report that the child failed any two critical items or any three items overall. In this study, parents completed the MCHAT questionnaire for 93 PT and 35 FT children at the 18-month assessment and for 93 PT and 30 FT children at the 24-month assessment. As the MCHAT is designed to be used between the ages of 16 and 30 months, it was not administrated at the 36-month assessment.

#### 2.2.2. Autism Diagnostic Observation Schedule

The ADOS [36] is a standardized assessment of communication, social interaction, and play or imaginative use of materials for diagnosing ASD. This semi-structured direct assessment of a child's social and communication skills and behavior comprises four modules based on verbal skills and is designed for use from 2 years to adulthood. The Toddler Module (ADOS-T; [37]) was designed for use with children younger than 30 months. We administrated the ADOS-T to 101 PT and 37 FT children at the 18-month assessment and to 97 PT and 37 FT children at the 24-month assessment. Module 1 was administered at the 36-month assessment to 93 PT and 33 FT children.

Items are scored based on observations of a child's behavior, with scores ranging from 0 (no abnormality) to 3 (moderate to severe abnormality). The ADOS-T and the ADOS algorithm provide ranges of concern that represent the severity of autism symptoms, namely, little-to-no, mild-to-moderate, and moderate-to-severe concern. Children whose algorithm scores are in the mild-to-moderate or moderate-to-severe concern range are classified as at risk for ASD, whereas children with algorithm scores in the little-or-no concern range are considered as not at risk for ASD.

#### 2.2.3. Autism Diagnostic Interview-Revised

The ADI-R [34] is a standardized, semi-structured, investigator-based interview for parents or caregivers of individuals referred for a possible ASD diagnosis. It provides a diagnostic algorithm for the ICD-10 [38] and DSM-IV (American Psychiatric Association, 1994) definitions of autism from early childhood to adult life. A toddler version of the ADI-R (the ADI-T) intended for children under 4 years of age is also available and was used in the current study [39]. It includes 125 items that address three domains of functioning (namely, language/communication, reciprocal social interactions, and restricted, repetitive, and stereotyped behaviors and interests) as well as other aspects of behaviors. Items also refer to the onset of ASD symptoms and general development.

For the majority of ADI-R items, scores of 0 to 3 are determined based on the type or quality of a specific behavior, as well as its frequency and severity. In this study, we used the new ADI-R algorithm for toddlers and young preschool children proposed by Kim and Lord [39]. This algorithm provides ranges of concern that represent the severity of autism symptoms, namely, little-to-no concern, mild-to-moderate concern, and moderate-to-severe concern. A child whose algorithm score is in the mild-to-moderate or moderate-to severe concern range is classified as at risk for ASD. In this study, we administrated the toddler version of the ADI-R to 96 PT and 36 FT children at the 24-month assessment. At the 36-month assessment we administered the SCQ (see below), which is based on the ADI-R. We did this to avoid burdening parents with the same long interview that they had completed only a year before, especially when there are no concerns regarding ASD risk. Thus, in cases of children who had an ADOS algorithm score above the autism spectrum cutoff at the 36-month assessment, the ADI-R was also completed in order to obtain a comprehensive picture of the children who were at risk for ASD.

#### 2.2.4. Social Communication Questionnaire

The SCQ [34] is a 40-item parent-report questionnaire that enquires about the autistic characteristics of individuals with a mental age of at least 2 years. It is based on the ADI-R [40]. Each item is scored 0 (typical development) or 1 (symptom of autism), and total scores range from 0 to 39 (as the first item is a language screening question that is not included in the total score). Two versions of the SCQ are available: Lifetime and Current. The Lifetime version yields a total score that is interpreted with reference to cutoff scores. Scores above the cutoff of 15 suggest that an individual is likely to have ASD and that a more extended evaluation should be undertaken. In this study, we administrated the SCQ to 92 parents of PT children and 33 parents of FT children at the 36-month assessment.

#### 2.2.5. Mullen Scales of Early Learning

The Mullen Scales of Early Learning (MSEL; [41]) assess the developmental functioning of children from birth through 68 months of age. The MSEL composite score offers a standardized general score (M = 100, SD = 15) based on four standardized scales (M = 50, SD = 10): fine motor, visual reception, expressive language, and receptive language; it also includes a gross motor scale. In this study, we administrated the MSEL to 101 PT and 37 FT children at the 18-month assessment, to 97 PT and 37 FT children at the 24-month assessment and to 94 PT and 33 FT children at the 36-month assessment.

#### 2.2.6. Clinical Judgment

At the 36-month assessment, clinical judgment of each child's diagnosis was done by well-trained professionals (NY, AHG, MY, and EF) based on the DSM-IV [1] criteria.

## 3. Results

**Figure 1:**
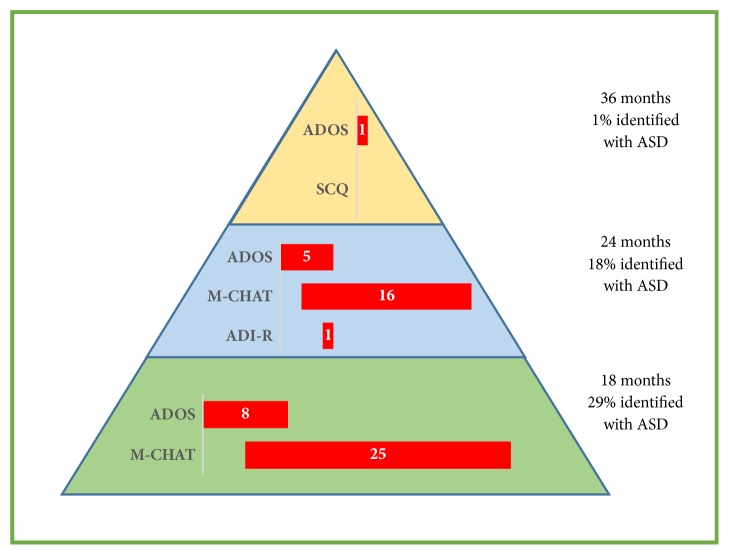
*Prevalence of *autism spectrum disorder* risk among preterm children*.* Note*. MCHAT: Modified Checklist for Autism in Toddlers, ADOS: Autism Diagnostic Observation Schedule, and ADI-R: Autism Diagnostic Interview-Revised.

At 18 months, the MCHAT and the ADOS-T were administered. As can be seen in Table 2 and Figure 1, twenty-five PT children (27%) screened positive on the MCHAT and eight children (8%) received ADOS-T algorithm scores that indicated an elevated ASD concern; only four children were identified by both the MCHAT and ADOS-T as being at risk for ASD. Among the FT children, three (9%) screened positive on the MCHAT and none had an ADOS-T algorithm score that indicated an elevated ASD concern.

At 24 months, the MCHAT, ADOS-T, and ADI-T were administered. Sixteen PT children (17%) screened positive on the MCHAT, five (5%) had ADOS-T algorithm scores that indicated an elevated ASD concern, and only one child (1%) was classified as having an ASD concern using the ADI-T. Whereas three children were identified by both the MCHAT and ADOS-T, only one child was identified by all three instruments. This child was also previously identified with ASD risk using the MCHAT and ADOS-T at the age of 18 months. Of the 18 children (18%) who were identified with ASD risk on one or more instruments at the age of 24 months, 14 were previously classified with ASD risk at the age of 18 months; the remaining 4 had previously scored within the norms on all instruments at the 18-month assessment. Among FT children, two (7%) screened positive on the MCHAT and none had ADOS-T or ADI-T algorithm scores that indicated an ASD concern. The two children who screened positive on the M-CHAT at the age of 24 months had previously scored within the norms on all instruments at 18 months.

At 36 months, the ADOS and the SCQ were administered. As assessed by clinical judgment and semi-structured developmental interviews with the parents, only one PT child (1%) was diagnosed with ASD. This child was the only child who had an ADOS algorithm score above the autism spectrum cutoff at the 36-month assessment. He was also identified with ASD risk at 18 and 24 months using the MCHAT, ADOS-T, and ADI-T. The ADI was conducted again at 36 months with the mother of this child and his score indicated ASD risk. None of the PT children screened positive on the SCQ. Only one FT child (3%) had an ADOS algorithm score that indicated ASD risk; he was not identified with ASD risk on any other ASD assessment measure at any time. The ADI-R was also conducted with the mother of this child at 36 months and his score was in the little-to-no concern range. Clinical judgments indicated that he had significant language difficulties and attention deficits, rather social-communication impairments. None of the FT children screened positive on the SCQ.

The sensitivity and the specificity of each measure were assessed at 18, 24, and 36 months among the PT group (see Table 3). As stated, the only child who was diagnosed with ASD was previously identified at 18 and 24 months with the MCHAT, ADOS-T, and ADI-T. Hence, the sensitivity of these measures in this sample was very high (100%). However, the specificity of the MCHAT and the ADOS-T was lower, indicating that at 18 and 24 months a high number of children who were identified with ASD risk (especially when using the MCHAT) did not receive a diagnosis of ASD at the 36-month assessment. The ADI-T specificity was very high (100%), indicating that only the child who was diagnosed eventually with ASD was identified with the ADI-T at 24 months. Since the best clinical practice is the combined use of ADOS and ADI for diagnosis of ASD [42], both tools were administrated at the assessment of the 24 months. The sensitivity and the specificity when both assessments were used together were also very high (100%), as with those of the ADI-T separately. The sensitivity of the SCQ administered at 36 months was very low (0%), as the one child who was diagnosed with ASD did not screen positive on the SCQ. The specificity, however, was very high (100%) since there were no children who screened positive with the SCQ. Yet, it is important to note that, due to the relative small sample size, we observed a low frequency of children diagnosed with ASD. Thus, the values of the sensitivity and specificity of the reported measures should be addressed and interpreted with caution.

Overall, 33 PT children and 6 FT children were identified with ASD risk at one or more of the assessments at the ages of 18 and/or 24 and/or 36 months. Among the PT children only one child, who was born at 33 GA weeks, was diagnosed at 36 months with ASD based on the information received from the parents, the ADOS at 36 months, the MCHAT, ADOS-T, and ADI-T at 18 and 24 months, and by clinical judgment at 36 months. Thus, we sought to deepen our understanding of the clinical characteristics of the 32 PT cases who were “false positives” (i.e., children who were identified with ASD risk using at least one of the ASD-related measures but who were not eventually diagnosed with ASD).* T-test* analyses were conducted to assess differences between the groups (children with positive screening versus children with negative screening) with regard to medical and developmental characteristics. As presented in Table 4, significant differences were found between the groups regarding gestational age, birth weight, and the MSEL scores. Next, a multilevel logistic regression was estimated with the predictors of GA, MSEL score, and age at assessment (i.e., 18, 24, or 36 months) to investigate if these variables can predict ASD risk. The age of assessment had no significant effect on the likelihood of ASD risk, but the GA and MSEL score were both significant predictors. Lower gestational age and lower MSEL scores were associated with higher likelihood of ASD risk among the PT group. Full model estimates are provided in Table 5.

## 4. Discussion

Growing public and clinical awareness exists in relation to the increased risk of ASD among PT children [23, 24, 28]. Furthermore, the early detection of ASD is significant for early intervention and optimal development. As such, the current prospective research aimed to evaluate the long-term risk for ASD among young PT children at 18, 24, and 36 months. Surprisingly, we detected lower rates of PT children with ASD than previously reported using observational instruments [23, 24]. In fact, at 36 months, comprehensive assessment using direct observations, parental reports, and clinical judgments led to only one child (1%) in the PT group who was diagnosed with ASD—which is similar to the rate of ASD found in the general population. This finding may suggest that the previously reported rates of ASD among PT children may have been overestimated, especially when parental reports served as the only source for risk assessment. Alternatively, the fact that most studies regarding ASD among PT children have included very PT children with a narrower GA range than in our study (i.e., 24–34 weeks) could also explain our lower rates of ASD among these children.

Another goal of the study was to examine the similarities and differences in the long-term risk for ASD employing parental reports compared to direct assessments by well-trained professionals. As expected, parent-completed MCHAT questionnaires yielded more at risk children than the ADOS administered by trained professionals at 18 and 24 months among both PT and FT children. The difference between employing parental reports compared to direct assessments is consistent with those of previous studies [23, 24]. Given that the MCHAT is intended to be used as a screening tool for determining whether further assessment is necessary, these results emphasize the potential risk of using the MCHAT questionnaire alone and indicate that caution should be taken when interpreting results from this questionnaire only, especially in cohorts of PT children. There has been considerable criticism of the sole use of the MCHAT [43–45], and it is important to note that a new version of the MCHAT with a follow-up interview, which improves the sensitivity and specificity, was validated in 2014 [46]. This later version was not available when we initiated the study.

With respect to the stability of ASD risk classifications among PT children, the rates of children who were identified with ASD risk decreased over time; this is in line with our previous findings in relation to 8-, 12-, and 18-month PT infants [26]. These findings demonstrate the challenge of identifying ASD risk earlier among young children (particularly in PT cohorts) and emphasize the importance of repeated assessments. The sensitivity of the ASD measures was relatively high at different ages, but the specificity was lower. The child who was diagnosed with ASD at 36 months was previously identified by all of the ASD measures, yet many children who were identified with ASD risk at 18 and 24 months did not maintain the diagnosis at 36 months. The ADI-T had a very high sensitivity and specificity. It was the most accurate measure for detecting the PT child who was eventually diagnosed with ASD. This child was the only one who classified with an ASD concern using the ADI-T at 24 months. Yet, it is important to note that, due to the relative small sample size, we observed a low frequency of children diagnosed with ASD. Thus, our values of the sensitivity and specificity of the measures should be addressed and interpreted with caution.

We also describe the stability of long-term risk for ASD among the PT group, in comparison to the FT group. So far this issue has been investigated only with parental reports, using the MCHAT questionnaire at 24 months [30, 47]. In congruence with previous research, at 18 and 24 months, we found increased rates of ASD risk, as determined by both parental reports and direct assessments, among young PT children in comparison to FT children. However, only one PT child was eventually diagnosed with ASD at 36 months. In other words, a difference in the rate of ASD risk between PT and FT children was yielded mainly at the young ages. It is possible that the higher risk for ASD found among young PT children compared to FT children reflects general developmental difficulties that are not specific to ASD, since it may be more difficult to distinguish between general developmental concerns and ASD specifically at the young ages.

Finally, among the 33 PT children who were identified with ASD risk at the ages of 18 and/or 24 months, only one child was eventually diagnosed with ASD at 36 months. That is, some PT children who were classified as having ASD concerns at an early age had later assessments that indicated little-to-no ASD-related concern. Our data revealed that earlier GA and lower general developmental abilities were associated with elevated ASD risk. Children who screened positive for ASD at 18 and 24 months but not diagnosed with ASD at 36 months were born earlier and had lower developmental scores on the MSEL than children who screen negative for ASD. These results again demonstrate the difficulty of differentiating between ASD and other developmental disorders and/or difficulties, especially at early ages among PT children. It is thus essential to take general characteristics of development and the unique difficulties associated with prematurity [16] into account when examining risk for ASD. Nonetheless, developmental disorders and/or difficulties are a common comorbidity in children with ASD [31].

The study's strength lies in its longitudinal design, which enabled us to examine the risk for ASD diagnosis over time. In addition, the inclusion of FT children made it possible to evaluate the long-term risk for ASD in a sample of PT children in comparison to FT children. Finally, we used both parental reports and direct assessments for all of the children, not only for those who screened positive for ASD through questionnaires. One limitation of our study is that it did not include the now available MCHAT follow-up interviews, which might have increase the specificity of the MCHAT. Additional challenges are the relatively low participation rate and the small sample size. In addition, the ADI-R was only completed at the 24-month assessment and not the 36-month assessment to avoid burdening the parents. Instead, the SCQ, a screening measure, was used to indicate the presence/absence of symptoms at this age. The SCQ is a screening measure and thus may be considered as less accurate for the purpose of diagnosing ASD risk. In future studies, it may be useful to examine different groups of PT children separately, according to gestational age or severity. It may also be interesting to examine additional variables that may distinguish between PT children with positive and negative ASD screenings, such as parent-child interaction, regulation abilities, and behavior characteristics, and to follow up the children later in childhood, when social demands increase and social difficulties may emerge.

## Figures and Tables

**Table 1:** Demographic and medical characteristics.

Characteristic	PT children	FT children	Group differences
	(n= 110)	(n= 39)	
Gender			
Male, *n* (%)	63 (57%)	21 (54%)	*ns*
Female, *n* (%)	47 (43%)	18 (46%)	
Gestational age (weeks)			
M (SD), range	31.16 (2.63), 24–34	39.82 (0.97), 37–41	PT < FT*∗*
≤ 28, *n* (%)	19 (17%)		
29-32, *n* (%)	34 (31%)		
33-34, *n* (%)	57 (52%)		
Birthweight (grams)			
M (SD), range	1556.02 (480.98), 490–2400	3372.56 (345.89), 2500–4258	PT < FT*∗*
1 min. Apgar score			
M (SD), range	7.65 (1.89), 1–9	8.95 (0.32), 7–9	PT < FT*∗*
5 min. Apgar score			
M (SD), range	8.9 (1.3), 2–10	9.9 (0.31), 9–10	PT < FT*∗*
Ventilation duration (days)			
M (SD), range	17.02 (34.85), 0–205	--	
Hospitalization duration			
M (SD), range	44.32 (36.70), 9–205	2.95 (0.89), 2–5	PT > FT*∗*
Maternal age at birth (years)			
M (SD), range	31.54 (5.86), 19–51	33.03 (4.18), 27–41	*ns*
Maternal education (years)			
High-school education *n* (%)	21 (19%)	4 (10%)	
Non-academic professional	19 (17%)	4 (10%)	
education *n* (%)	46 (42%)	22 (57%)	*ns*
Undergraduate degree *n* (%)	24 (22%)	9 (23%)	
Master's and/or doctoral			
degree *n* (%)			
Income			
Below median *n* (%)	23 (21%)	3 (8%)	
Median range *n* (%)	71 (64%)	28 (72%)	*ns*
Above median *n* (%)	16 (15%)	8 (20%)	

*∗*p < .01.

**Table 2:** Autism spectrum disorder *risk among preterm and full- term children*.

		PT children	FT children
18 months	M-CHAT, *n* (%)	25 (27%)	3 (9%)

	ADOS, *n* (%)	8 (8%)	0

24 months	M-CHAT, *n* (%)	16 (17%)	2 (7%)

	ADOS, *n* (%)	5 (5%)	0

	ADI-R, *n* (%)	1 (1%)	0

36 months	ADOS, *n* (%)	1(1%)	1 (3%)

	SCQ, *n* (%)	0	0

*Note*. MCHAT: Modified Checklist for Autism in Toddlers, ADOS: Autism Diagnostic Observation Schedule, and ADI-R: Autism Diagnostic Interview-Revised.

**Table 3:** *Sensitivity and specificity of the* autism spectrum disorder *measures*.

		Sensitivity (%)	Specificity (%)
18 months	M-CHAT	100	72.8
	ADOS-T	100	93

24 months	M-CHAT	100	83.7
	ADOS-T	100	95.8
	ADI-T	100	100

36 months	SCQ	0	100

*Note*. MCHAT: Modified Checklist for Autism in Toddlers, ADOS: Autism Diagnostic Observation Schedule, and ADI-R: Autism Diagnostic Interview-Revised.

**Table 4:** Medical and developmental characteristics of preterm children with positive versus negative screening for autism spectrum disorder.

	Children with positive screening	Children with negative screening	Group differences
Gestational age (weeks) (n=100)			
M (SD), range	30.34 (2.77), 24.71-34	31.74 (2.35), 24.29-34	PS < NS*∗*

Birth weight (grams) (n=100)			
M (SD), range	1352.16 (470.46), 510-2284	1661.22 (444.23), 490-2400	PS < NS*∗∗*

MSEL 18 months (n=100)			
M (SD), range	88.44 (10.98), 70-122	98.72 (11.77), 69-126	PS < NS*∗∗*

MSEL 24 months (n=96)			
M (SD), range	95.94 (14.06), 61–118	109.81 (14.51), 65–139	PS < NS*∗∗*

MSEL 36 months (n=94)	103.03 (18.58), 49–137		
M (SD), range		115.03 (13.47), 70–141	PS < NS*∗∗*

*Note*. MSEL: Mullen Scales of Early Learning, PS: positive screening, and NS: negative screening. *∗*p <. 05, *∗∗p < .01*.

**Table 5:** Risk for autism spectrum disorder among preterm children.

	B	Se	Z	p	Odds ratio
Intercept	17.95	3.51	5.10	.00	
GA (weeks)	-.65	.00	-502.4	.00	.52
MSEL composite score	-.08	.00	-61.2	.00	.92
Age of assessment (18, 24, and 36 months)	.67	.63	1.10	.29	1.95

*Note*. GA: gestational age and MSEL: Mullen Scales of Early Learning.

## Data Availability

The data used to support the findings of this study are available from the corresponding author upon request.
